# Cognitive Control of Episodic Memory in Schizophrenia: Differential Role of Dorsolateral and Ventrolateral Prefrontal Cortex

**DOI:** 10.3389/fnhum.2015.00604

**Published:** 2015-11-10

**Authors:** John D. Ragland, Charan Ranganath, Joshua Phillips, Megan A. Boudewyn, Ann M. Kring, Tyler A. Lesh, Debra L. Long, Steven J. Luck, Tara A. Niendam, Marjorie Solomon, Tamara Y. Swaab, Cameron S. Carter

**Affiliations:** ^1^Department of Psychiatry and Behavioral Sciences, University of California, DavisSacramento, CA, USA; ^2^Department of Psychology, University of California, DavisDavis, CA, USA; ^3^Center for Neuroscience, University of California, DavisDavis, CA, USA; ^4^Department of Psychology, University of California, BerkeleyBerkeley, CA, USA; ^5^Center for Mind and Brain, University of California, DavisDavis, CA, USA

**Keywords:** schizophrenia, episodic memory, cognitive control, functional magnetic resonance imaging, recollection, familiarity

## Abstract

**Background:** Dorsal (DLPFC) and ventral (VLPFC) subregions in lateral prefrontal cortex play distinct roles in episodic memory, and both are implicated in schizophrenia. We test the hypothesis that schizophrenia differentially impairs DLPFC versus VLPFC control of episodic encoding.

**Methods:** Cognitive control was manipulated by requiring participants to encode targets and avoid encoding non-targets based upon stimulus properties of test stimuli. The more automatic encoding response (target versus non-target) was predicted to engage VLPFC in both groups. Conversely, having to overcome the prepotent encoding response (non-targets versus targets) was predicted to produce greater DLPFC activation in controls than in patients. Encoding occurred during event-related fMRI in a sample of 21 individuals with schizophrenia and 30 healthy participants. Scanning was followed by recognition testing outside the scanner.

**Results:** Patients were less successful differentially remembering target versus non-target stimuli, and retrieval difficulties correlated with more severe disorganized symptoms. As predicted, the target versus non-target contrast activated the VLPFC and correlated with retrieval success in both groups. Conversely, the non-target versus target contrast produced greater DLPFC activation in controls than in patients, and DLPFC activation correlated with performance only in controls.

**Conclusion:** Individuals with schizophrenia can successfully engage the VLPFC to provide control over semantic encoding of individual items, but are specifically impaired at engaging the DLPFC to main context for task-appropriate encoding and thereby generate improved memory for target versus non-target items. This extends previous cognitive control models based on response selection tasks to the memory domain.

## Introduction

Deficits in lateral prefrontal cortex (PFC) control of encoding and retrieval play a prominent role in episodic learning and memory impairments in schizophrenia (SZ; Achim and Lepage, 2005; Ranganath et al., 2008). Prefrontal dysfunction was first suspected on list-learning tasks such as the California Verbal Learning test when individuals with SZ failed to generate semantic organizational strategies to facilitate encoding and retrieval (Iddon et al., 1998; Stone et al., 1998; Roofeh et al., 2006), but benefitted when strategies were provided (McClain, 1983; Ragland et al., 2003; Bonner-Jackson et al., 2005). Functional neuroimaging further implicated the PFC. For example, an fMRI meta-analysis (Ragland et al., 2009), found that the most consistent reductions in task-related activation in schizophrenia during encoding and retrieval were in the lateral PFC. Moreover, PFC dysfunction and associated memory deficits were reduced when cognitive control demands were minimized by providing incidental encoding strategies (Bonner-Jackson et al., 2005; Ragland et al., 2005, 2009). However, the lateral prefrontal cortex is composed of functionally and anatomically distinct sub-regions (D’Esposito et al., 1999; Petrides, 2000; Petrides et al., 2002), and the regional specificity of these episodic memory related PFC deficits in SZ is not well established. Establishing this regional specificity could help to alleviate memory dysfunction by identifying functional networks that could be bootstrapped through cognitive training, as well as pinpointing dysfunctional networks that can be targeted for development of new treatments.

Anatomical studies in humans and non-human primates support a dorso-ventral division within lateral PFC, with the dorsolateral subregion (DLPFC) forming indirect reciprocal connections with hippocampus through retrosplenial and parahippocampal cortices (Goldman-Rakic et al., 1984; Morris et al., 1999; Petrides and Pandya, 1999), and the ventrolateral subregion (VLPFC) establishing connections via perirhinal cortex (Petrides and Pandya, 2002). fMRI research also supports functional dissociations within memory paradigms, with DLPFC activation associated with organizational processing and manipulation and monitoring of goal-relevant relational information, and VLPFC activation associated with selection and maintenance of goal-relevant item information (Blumenfeld et al., 2013). Therefore, the purpose of this study was to interrogate this dorsal/ventral subdivision by utilizing an fMRI episodic memory paradigm that manipulates cognitive control demands during word list learning.

Cognitive control manipulations were based upon the guided activation model (Miller and Cohen, 2001), which states that the DLPFC represents and maintains context for responding – or goals, which, in turn, biases processing in posterior and pre-motor areas to support task appropriate responding. To date, this model has been examined in SZ using response selection tasks such as the Stroop (Perlstein et al., 1998; Barch et al., 2004) and AX-CPT (Barch et al., 2001; MacDonald and Carter, 2003), which found specific impairments in SZ that were not due medication (Braver et al., 2001; MacDonald et al., 2005), were linked to reduced DLPFC activation (MacDonald and Carter, 2003) and fronto-parietal connectivity (Yoon et al., 2008), and associated with global functioning and severity of disorganized symptoms (Barch et al., 2003; Yoon et al., 2008). In the current study, we translated these response selection paradigms into the episodic memory domain through development of the Context Maintenance Encoding Task (CMET).

**FIGURE 1:**
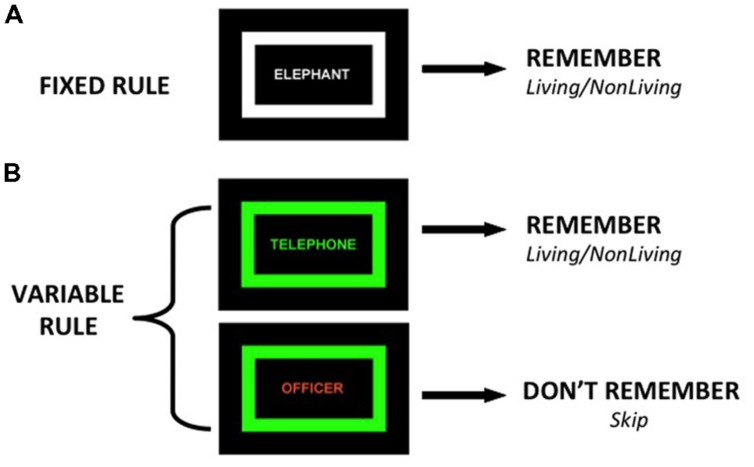
**Illustration of two encoding conditions: (A) Fixed Rule – participants make living/non-living judgment and try to remember all items, (B) Variable Rule – when color of frame and word match (target trial) participants make semantic judgment and try to remember the items, if colors do not match (non-target trial) participants skip word and do not try to remember**.

During the CMET, participants encode words during two tasks. During the fixed rule task, participants view a list of words in which the color of the words and a surrounding rectangular frame is identical (**Figure 1**), and make a two-button semantic “living”/“non-living” response for every word and try to remember it for a subsequent recognition task. The fixed rule task was designed as a baseline task in which no group differences are predicted, given evidence that providing a semantic encoding task reduces group differences in word recognition and normalizes VLPFC function (Ragland et al., 2003, 2005; Bonner-Jackson et al., 2005). During the variable rule task, the color of the word and surrounding frame is identical on some trials (targets) and different on other trials (non-targets). Participants are told to remember the word and make a “living”/“non-living” response only if the word is a target. If the word is a non-target, they are instructed not to try to remember the word and use their other hand to make a “skip” response, as their memory for that item will not be tested. Participants, however, are tested on all words from both conditions.

In this variable rule design, contrasting non-targets minus targets is expected to produce increased DLPFC activation in healthy controls (HC), as increased cognitive control is required to overcome the prepotent response to encode test stimuli. This prepotent encoding response is created because the majority of trials across conditions (67%) include target items. As in the fixed rule condition, the contrast of target minus non-target trials is expected to reveal increased VLPFC activation because of greater semantic encoding demands for target versus non-target items. Performance on the variable rule task is predicted to show a group by condition interaction, reflecting less selective encoding of target versus non-target stimuli in the patient group. During fMRI, patient impairments are predicted for contrasts emphasizing DLPFC versus VLPFC control.

To date, we are not aware of any previous neuroimaging studies that have examined processing of target versus non-target information during an episodic encoding task in patients with SZ. However, there are a number of behavioral studies that examined the interaction between executive control and episodic memory in SZ utilizing a directed forgetting (DF) paradigm (Johnson, 1994; MacLeod, 1998). In the common variant of the DF task, participants are presented with a list of items to encode, and after each item, a cue appears to indicate whether that item is to be remembered (TBR) or to be forgotten (TBF). This produces a DF effect in healthy volunteers (i.e., better memory for TBR than TBF) that is reduced in patients with temporal lobe seizure disorders (Fleck et al., 1999) and right frontal lesions (Conway and Fthenaki, 2003). Although encoding and retrieval methods varied, all three previous SZ studies found a group by cue type interaction, with a reduced DF effect in patients versus controls (Sonntag et al., 2003; Menon et al., 2004; Müller et al., 2005). This is the same performance interaction that we expect in the current study for the variable rule task.

## Materials and Methods

### Participants

Data were acquired on 28 individuals with SZ and 30 HC. Data were excluded for one HC and one SZ with excessive movement (i.e., more than 3 mm), and five SZ and two HC with below chance task performance, leaving a final sample of 21 SZ and 30 HC. Individuals were matched at the group level for age, gender, handedness, and parental education, but participant education was lower in the patient sample (**Table 1**). Illness onset often disrupts educational attainment and we, therefore, followed recommended procedures (Resnick, 1992) of matching groups on parental rather than participant education. There were no differences in age, gender distribution, or handedness between individuals with SZ who were and were not included in data analysis. The study was approved by the institutional review board of the University of California at Davis and informed consent forms were signed by all participants.

**Table 1:** Participant demographics.

	Healthy control group (*n* = 30)		Patients with schizophrenia (*n* = 21)		
	Mean	*SD*	Mean	*SD*	*p*-value
Age (years)	23.9	5.2	25.3	8.1	ns
Gender (% male)	80.0		76.2		ns
Handedness (% right)	90.0		85.7		ns
Education (years)	15.1	2.6	13.1	10.0	<0.05
Parental Education (years)	14.8	2.4	15.6	2.6	ns
SANS (total)			39.1	16.8	
SAPS (total)			14.9	17.1	
BPRS (total)			40.6	8.7	

*SANS, Scale for the Assessment of Negative Symptoms; SAPS, Scale for the Assessment of Positive Symptoms; BPRS, Brief Psychiatric Rating Scale; SD, standard deviation; ns, no significant group difference at p < 0.05, two-tailed.*

The Structured Clinical Interview for DSM-IV-TR (SCID-I) confirmed the diagnosis of SZ, and verified that HC were free of lifetime history of Axis I disorder. The SCID-I was conducted by Masters or Doctoral level clinicians and confirmed by consensus conference. Symptoms were measured using the Scale for the Assessment of Negative Symptoms (SANS; Andreasen, 1983), Scale for the Assessment of Positive Symptoms (SAPS; Andreasen, 1984), and Brief Psychiatric Rating Scale (BPRS; Ventura et al., 1993). Following previous work (Barch et al., 2003), positive (POS), negative (NEG), and disorganization (DISORG) symptom dimensions were computed from the SANS, SAPS, and BPRS. The Global Assessment of Functioning (GAF; American Psychiatric Association, 1994) estimated overall psychological, social, and occupational functioning. Patients were early in their illness (mean ±*SD* = 5.2 ± 2.8 years since illness onset) and clinically stable. All but one patient was receiving medication (1 typical, 17 atypical, 2 anxiolytic). Exclusion criteria were: WASI IQ < 70, drug or alcohol abuse or dependence in the previous 3 months (confirmed by urinalysis), major medical or neurological illness, significant head trauma, or any known MRI contraindication. Data collection began after participants provided written informed consent following Institutional Review Board approval.

### Stimuli and Design

Stimuli consisted of 600 words from the MRC Psycholinguistic Database^1^ (Coltheart and Evans, 1981). All stimuli were nouns, averaging six letters long (range 3–13), with above average imageability (mean = 557; range 339–659) and concreteness (mean = 563; range 381–654). Three hundred and sixty words were randomly assigned to encoding and the remaining 240 served as foils for recognition testing. Encoding was performed during MRI and recognition testing was administered after scanning was complete.

During encoding, stimuli were visually presented in the middle of a black background for 2 s, with a variable inter-stimulus-interval (ISI) averaging 4 s (range 2–14 s.) during which a fixation cross was present. Stimuli were presented in a colored font (white, orange or green) surrounded by a rectangular frame of one of the same three colors (**Figure 1**). Encoding was administered over six 4-min fMRI runs of three blocks each. Each run alternated one fixed rule block (20 trials) with two variable rule blocks (10 target, 10 non-target trials), and the order of targets and non-targets were pseudo-randomized within the variable rule blocks. The order of runs across subjects was counter-balanced. Encoding conditions were as follows:

#### Fixed Rule

During this task (120 trials), the color of the word and surrounding frame was always white, and participants were instructed to use their right hand to make a two-button “living/non-living” judgment for each word and try to remember it because their memory would be tested. Participants were instructed to work as quickly and accurately as possible and guess if unsure.

#### Variable Rule

This task consisted of two trial-types; target and non-target. During target trials (120 trials), the color of the word and surrounding frame was identical (both green or both orange) and participants were instructed to make a “living/non-living” judgment and to remember the word because their memory would be tested. On non-target trials (120 trials), the color of the word and surrounding frame did not match and participants were instructed to use their opposite hand to make a one-button “skip” response and avoid making a living/non-living judgment or trying to remember the word because their memory would not be tested.

Following scanning, participants were de-briefed to explain that memory for all words (i.e., target and non-target) would be tested. The recognition task consisted of all 360 words seen during encoding (“old”) and 240 “new” unstudied foils. Participants were instructed to indicate whether each word was “old” (left hand response) or “new” (right hand response)” and indicate level of confidence with a three-button response (i.e., 3 = high, 2 = medium, 1 = low). Participants were given practice tasks, and were instructed on the importance of using the full range of confidence ratings. Recognition testing was self-paced.

### MRI Acquisition

Data were obtained at the UC Davis Imaging Research Center on a 3-T Siemens Tim Trio scanner (Erlangen, Germany) with a Siemens 8 channel phased array coil. After acquiring a rapid 3-plane localizer, *trans*-axial T2 weighted images were acquired with spatial resolution of 0.7 mm × 0.4 mm × 3.4 mm. A reference echo-planar point spread function sequence with spatial resolution of 3.4 mm × 3.4 mm × 3.4 mm was used for motion and distortion correction of the subsequent functional images. Functional images were acquired with blood oxygenation level dependent imaging (BOLD) using a 34-slice whole-brain, single-shot gradient-echo echo-planar sequence (TR 2000 ms, TE 25 ms, flip angle 90°, FOV 220 mm × 220 mm, slice thickness 3.4 mm, no gap). The sequence of slice acquisition was inter-leaved (odd then even, bottom to top).

### Data Processing and Analysis

#### Behavioral Data

To assess engagement during encoding, living/non-living responses and reaction times were recorded and the number of non-responses and median reaction times (in ms.) were calculated. Because living/non-living judgments were often equivocal (e.g., “apple”) and because participants were engaged in semantic decision making regardless of response, response accuracy was not calculated. Number of non-responses and median reaction times were examined separately for the fixed rule and variable rule tasks using repeated-measures Analysis of Variance (ANOVA) to determine effects of group (SZ, HC) and, if applicable, trial type (target, non-target), as well as any group by trial type interactions.

For each task, recognition was examined for overall recognition accuracy (d′ = normalized hit rate – normalized false alarm rate), and for recollection (R) and familiarity (F) estimates by entering the 6-point confidence ratings into a receiver operator characteristic (ROC) analysis (Yonelinas, 1994). The three performance variables (d′, R, F) were entered into separate repeated-measures ANOVAs to determine effects of group and, if applicable, trial type and group by trial type interactions. *Post hoc* univarate ANOVAs were used to examine any higher-level interactions. Because not all variables were normally distributed, Spearman’s rank order correlation coefficients were used to measure associations between recognition performance and GAF and POS, NEG and DISORG symptoms. A two-tailed alpha level of 0.05 was used for significance testing, and all analyses were performed with SAS Version 9.2 (SAS Institute Inc., Cary, NC, USA).

#### MRI Data

Data were preprocessed using Statistical Parametric mapping (SPM8) including slice time correction, realignment to the median image, normalization to template space, and spatial smoothing (8 mm FWHM). Subject-level fMRI analysis was performed using the general linear model (GLM) in VoxBo^2^ 1.8. BOLD responses during fixed and variable rule encoding conditions were modeled by convolving vectors of predicted neural activity corresponding to each trial type with a canonical hemodynamic response function. Separate covariates were included to model response and non-response trials, but only response trials were examined in second-level analyses. Target and non-target trials were also modeled separately for the variable rule condition. Nuisance covariates were orthogonalized to the design matrix, and included global signal changes, trial-specific shifts in baseline signal between scans, motion spikes, and an intercept. The design matrix also included a time-domain representation of low frequency (1/f) power and filters to remove frequencies >0.25 Hz and <0.02 Hz.

Parameter estimates from first-level GLM analyses for variable rule trials were entered into second-level one-sample and two-sample *t*-tests in SPM8. For the Fixed Rule condition there was one contrast (correct minus incorrect trials), and for the Variable Rule condition, there were two contrasts of interest (target minus non-target, and non-target minus target). Because hypotheses concerned the PFC, search space was restricted with a frontal lobe mask from the WFU_PickAtlas (Maldjian et al., 2003), and a *p* < 0.05 cluster-level correction for multiple comparisons was established with AlphaSim using a voxel-wise threshold of *p* < 0.005 and extent threshold of 17 voxels. This PFC mask combined Brodmann areas (BA) comprising DLPFC (BA 9, 46) and VLPFC (BA 44, 45, 47) cortex. Mean beta values for functional regions of interest (ROIs) showing above-threshold activity across all participants in the PFC were calculated and used to test for effects of group, encoding condition, and group by condition interactions using repeated-measures ANOVA. Robust linear regression (Yohai, 1987) was used to test the ability of task-related changes in these PFC beta values to predict task-related changes in recognition performance. Fisher’s *z* test was used to test for any group differences in regression results. In SZ, Spearman’s rank order correlations were also used to examine relationships between task-related changes in PFC beta values and GAF and POS, NEG, and DISORG symptoms.

## Results

### Behavioral Performance

#### Encoding

Participants responded on over 98% of trials, with no group difference in response rates for the fixed rule task [*F*(1,48) = 1.67, *p* = 0.20], or the variable rule task [*F*(1,48) = 0.001, *p* = 0.95]. On the variable rule task, there was no effect of trial type [*F*(1,48) = 0.80, *p* = 0.37] or any trial type interaction for response rates [*F*(1,48) = 0.43, *p* = 0.51]. Evaluation of median reaction times revealed that people with SC were, on average, 203 ms slower on both the fixed rule task [*F*(1,48) = 11.96, *p* < 0.01] and the variable rule task [*F*(1,48) = 9.24, *p* < 0.01]. Reaction times were also longer for target than non-target stimuli on the variable rule task for both groups [*F*(1,48) = 218.91, *p* < 0.0001], with no group by trial type interaction [*F*(1,48) = 3.11, *p* = 0.08]. Thus, although people with SZ had slower response times, they did not differ in response rates, and non-responses were low in both groups, reflecting full task engagement.

#### Recognition

**FIGURE 2:**
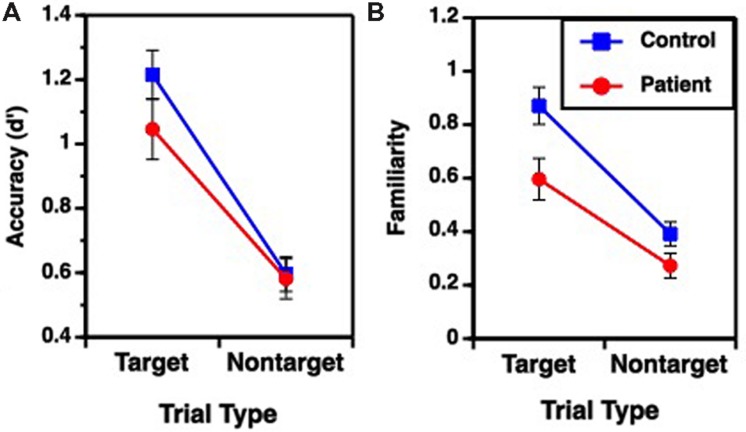
**Mean (±SEM) performance for target and non-target items during Variable Rule condition; (A) overall accuracy performance, (B) familiarity based retrieval.** No group performance differences were observed for Fixed Rule condition or for recollection based retrieval.

Performance on the recognition task was evaluated for overall accuracy (d′), and for familiarity (F) and recollection (R) estimates (**Table 2**), using separate one-way (fixed rule) or two-way (variable rule) ANOVAs. For the fixed rule task, there were no differences between groups in overall recognition accuracy [*F*(1,49) = 1.89, *p* = 0.17; Cohen’s *d* = 0.006]. This is consistent with previous studies demonstrating relatively intact item recognition in individuals with SZ when they are provided with a semantic encoding strategy (Bonner-Jackson et al., 2005; Ragland et al., 2005). For the variable rule task, there was a group by trial type interaction [*F*(1,49) = 4.07, *p* < 0.05] as well as a main effect of trial type [*F*(1,49) = 203.85, *p* < 0.0001]. As can be seen in **Figure 2A**, this interaction was due to a greater difference in recognition memory for target than for non-target items in HC than in people with SZ (Cohen’s *d* = 0.58), despite a lack of group differences for either target [*F*(1,49) = 1.98, *p* = 0.16] or non-target items [*F*(1,49) = 0.04, *p* = 0.85].

**Table 2:** Recognition task performance for participants in the control group and for schizophrenia patients.

	Healthy control group (*n* = 30)		Patients with schizophrenia (*n* = 21)		
	Mean	*SD*	Mean	*SD*	*p*-value
**Fixed rule**					
Accuracy (*d′*)	1.44	0.51	1.24	0.48	ns
Familiarity (F)	0.93	0.54	0.83	0.38	ns
Recollection (R)	0.38	0.21	0.32	0.22	ns
**Variable rule target**					
Accuracy (*d′*)	1.22	0.42	1.05	0.43	ns
Familiarity (F)	0.87	0.38	0.60	0.36	<0.05
Recollection (R)	0.29	0.19	0.31	0.18	ns
**Variable rule non-target**					
Accuracy (*d′*)	0.60	0.30	0.58	0.28	ns
Familiarity (F)	0.39	0.24	0.27	0.21	ns
Recollection (R)	0.14	0.11	0.16	0.10	ns

*Standard deviations are presented in the parentheses. SD, standard deviation; ns, no significant group difference at p < 0.05, two-tailed.*

Receiver operator characteristic analysis yielded and good model fit in both groups when R and F were estimated (sum of square of errors = 0.001). For the fixed rule task, there were no differences between groups [*F*(1,49) = 0.58, *p* = 0.45; Cohen’s *d* = 0.22] in familiarity performance. The variable rule task showed a group by trial type interaction [*F*(1,49) = 4.04, *p* < 0.05] and main effects of group [*F*(1,49) = 6.21, *p* < 0.05] and trial type [*F*(1,49) = 107.64, *p* < 0.0001] on familiarity performance. This is shown in **Figure 2B**, illustrating that participants with SZ were again less successful than HC in using the rule to constrain memory for target versus non-target items (Cohen’s *d* = 0.74). This interaction reflected a significant group difference for target items [*F*(1,49) = 6.76, *p* < 0.05], but only a trend-level group difference for non-target items [*F*(1,49) = 3.28, *p* = 0.08].

There were no recollection differences between groups for either the fixed rule task [*F*(1,49) = 1.13, *p* = 0.29; Cohen’s *d* = 0.29], or for the variable rule task for either target [*F*(1,49) = 0.14, *p* = 0.70; Cohen’s *d* = 0.11] or non-target items [*F*(1,49) = 0.37, *p* = 0.55; Cohen’s *d* = 0.19]. Recollection was, therefore, not included in subsequent analyses. In sum, examination of retrieval revealed that individuals with SZ had difficulty using the variable rule to promote goal-relevant memory for target versus non-target stimuli that was apparent in their overall accuracy and familiarity-based rather than recollection-based retrieval.

#### Clinical Correlations

As seen in **Table 3**, recognition accuracy was negatively correlated with the severity of disorganized symptoms in people with SZ. Individuals with better performance were less disorganized. No correlations were obtained with positive or negative symptoms or with estimates of global functioning (GAF).

**Table 3:** Spearman correlations between clinical dimensions and recognition accuracy.

	Global assessment of functioning	Positive	Disorganization	Negative
**Variable rule target *d*′**
*r*-value	0.24	–0.01	–0.43	–0.15
*p*-value	ns	ns	<0.05	ns
**Variable rule non-target *d*′**
*r*-value	0.29	–0.23	–0.61	–0.05
*p*-value	ns	ns	<0.005	ns
**Fixed rule *d*′**
*r*-value	0.22	–0.13	–0.47	0.06
*p*-value	ns	ns	<0.05	ns

*ns, not significant.*

### fMRI Results

Images showed little motion across x, y and z dimensions (mean = 1.36 ± 0.94 mm translational and 0.02 ± 0.02 degrees of rotational motion), and no differences between groups in translational [*F*(1,49) = 2.5, *p* = 0.12] or rotational movement [*F*(1,49) = 1.4, *p* = 0.24].

As predicted, when the fixed rule task was examined (correct minus incorrect trials), there were no significant differences in either frontal lobe region between individuals with and without SZ (Supplementary Figure S1). Therefore, remaining analyses focus on variable rule task.

**FIGURE 3:**
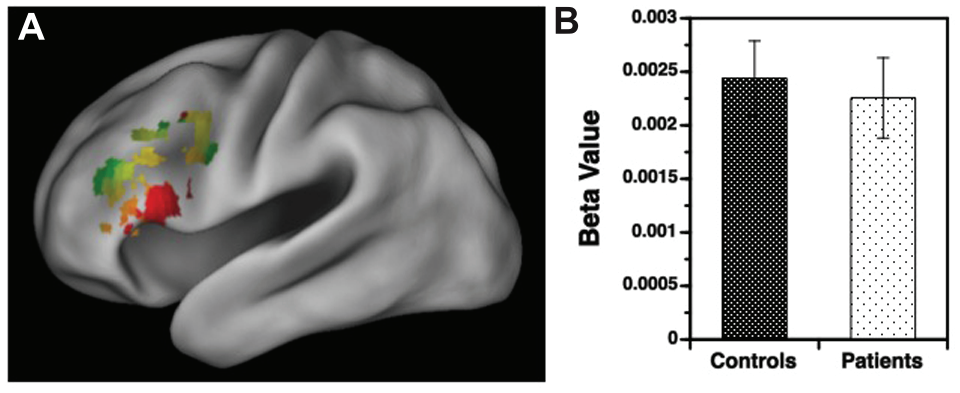
**(A)** Surface rendering of left hemisphere activation in the ventrolateral prefrontal cortex (VLPFC) during response to target versus non-target items during the variable rule condition. Above-threshold activity (*p* < 0.05, cluster corrected) is indicated in red for healthy controls (HC) and green for patients with schizophrenia. Areas of overlap between groups are indicated in yellow. **(B)** Corresponding regression coefficients (beta values) showing increased VLPFC activity for target versus non-target stimuli in patients and controls, with no group difference in VLPFC activation.

**FIGURE 4:**
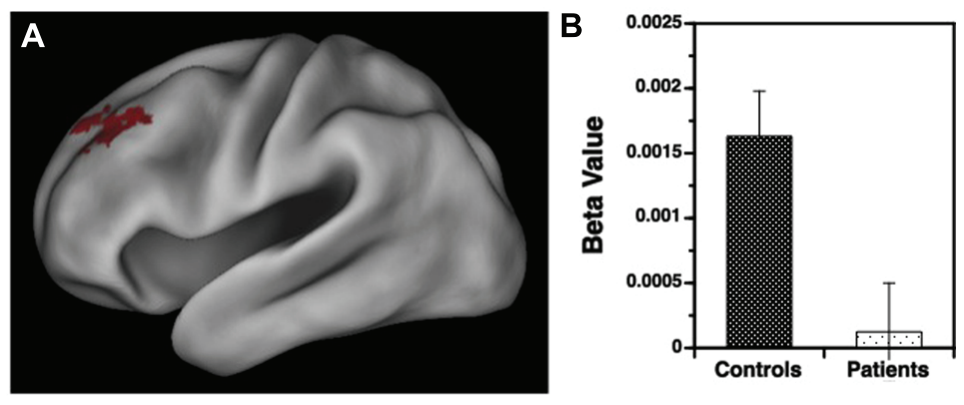
**(A)** Surface rendering of left hemisphere activation in the dorsolateral prefrontal cortex (DLPFC) during response to non-target versus target items during the variable rule condition. Above-threshold activity (*p* < 0.05, cluster corrected) is indicated in red for HC. No above-threshold activity was observed for the patient sample. **(B)** Corresponding regression coefficients (beta values) showing greater DLPFC activation in HC than in patients with schizophrenia for processing of non-target versus target items.

Results for the variable rule task are illustrated in **Figures 3** and **4**. As can be seen in **Figure 3A**, both groups showed left VLPFC activation in response to increased semantic encoding demands during target versus non-target trials. However, when increased cognitive control was needed to overcome the tendency to encode items during non-target versus target trials, there were predicted left hemispheric increases in DLPFC activation in the HC but not in individuals with SZ (**Figure 4A**).

#### Group Differences

Examination of left hemisphere VLPFC and DLPFC beta values generated by the contrasts of target and non-target conditions revealed a three-way interaction between trial type, region of interest (ROI), and group [*F*(1,49) = 12.88, *p* < 0.001]. There were also main effects of trial type [*F*(1,49) = 14.21, *p* < 0.001] and ROI [*F*(1,49) = 74.01, *p* < 0.0001], and an interaction between trial type and ROI [*F*(1,49) = 187.81, *p* < 0.0001]. To better understand these interactions VLPFC and DLPFC regions were examined separately. For the VLPFC, there were main effects of trial type [*F*(1,49) = 84.36, *p* < 0.0001], but no differences between groups [*F*(1,49) = 0.04, *p* = 0.84] or any group by trial type interaction [*F*(1,49) = 0.13, *p* = 0.72]. As seen in **Figure 3B**, VLPFC activity increased for target versus non-target trials equally in both groups. However, when DLPFC was examined, there was a group by trial type interaction [*F*(1,49) = 14.75, *p* < 0.001] and a main effect of trial type [*F*(1,49) = 20.04, *p* < 0.0001]. As can be seen in **Figure 4B**, this interaction was due to a greater increase in DLPFC activity for non-target versus target trials in HC than in individuals with SZ.

#### fMRI Relationships with Performance and Clinical Symptoms

**FIGURE 5:**
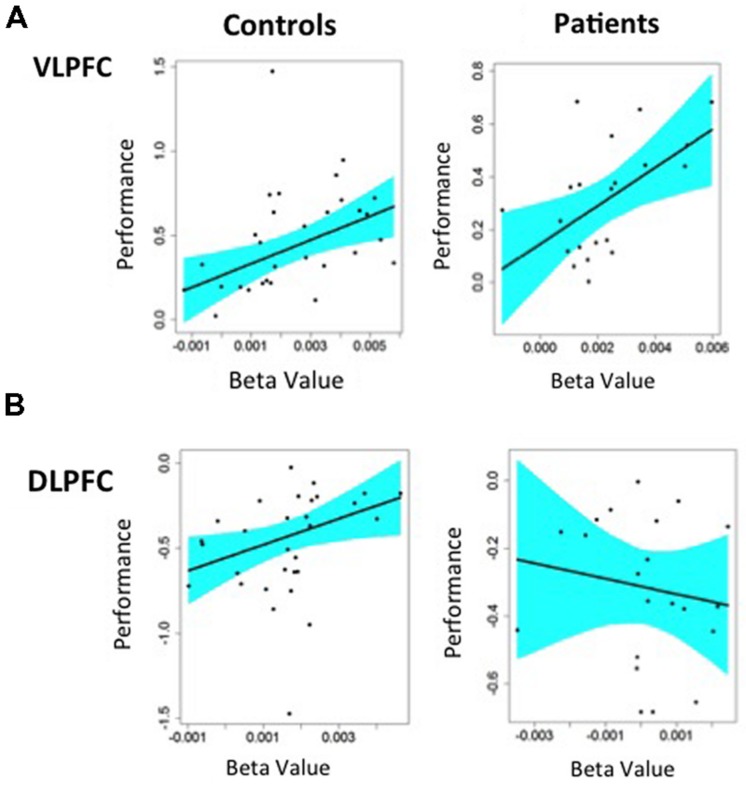
**Regression analysis predicting changes in Familiarity performance from changes in BOLD fMRI in left hemisphere prefrontal ROIs: (A) Greater VLPFC activity predicted better task performance for target minus non-target items in both HC and patients with schizophrenia, (B) Greater DLPFC activity predicted better task performance for non-target minus target item in HC.** Patients with schizophrenia showed a negative relationship between DLPFC activation and task performance that was not statistically significant.

We first examined the ability of target minus non-target activity in the left VLPFC to predict consequent changes in recognition performance. As seen in **Figure 5A**, increased VLPFC activity predicted increased familiarity for target versus non-target trials in HC (slope = 70.6 ± 23.7, *p* < 0.005) and in individuals with SZ (slope = 72.2 ± 27.1, *p* < 0.01), with no group difference in the strength of this fMRI/performance relationship (Fisher’s *z* = 0.22, *p* = 0.99). Thus, regardless of group membership, participants with greater VLPFC increases between target and non-target trials also showed greater recognition increases between these two trial types. We next tested whether increases in left DLPFC activity from non-target to target trials predicted increases in familiarity between non-targets and targets. As seen in **Figure 5B**, modulation of DLPFC activity predicted differences in task familiarity performance in HC (slope = 76.6 ± 34.1, *p* < 0.05), but not in people with SZ (slope = –22.6 ± 38.6, *p* = 0.66). When the strength of these correlations were tested, there was a significant difference between groups (Fisher’s *z* = 1.64, *p* < 0.05). A similar, but less robust, pattern of regression results were observed when changes in VLPFC and DLPFC activity were used to predict changes in overall recognition accuracy (*d*′).

Finally, Spearman correlations were used to test if changes in left VLPFC or DLPFC activity were associated with severity of positive, negative, or disorganized symptom dimensions. This did not reveal any significant correlations in individuals with SZ.

## Discussion

This study assessed regional specificity of lateral PFC dysfunction along the dorso-ventral axis in individuals with SZ during encoding of episodic memories. As in previous word recognition studies (Bonner-Jackson et al., 2005; Ragland et al., 2005), when SZ participants were provided with semantic encoding strategies (make a “living/non-living” judgment), and cognitive control demands were low (i.e., fixed rule task), item recognition was unimpaired, with no group differences in overall accuracy (*d*′), familiarity (F), or recollection (R). In contrast, when cognitive control demands were increased by requiring participants to encode only goal-relevant stimuli (i.e., targets), and overcome pre-potent tendencies to encode test stimuli when presented with non-targets, individuals with SZ were less successful than HC at selective encoding, and this was true both for d′ and F estimates. Moreover, performance success on this variable rule task was associated with less severe disorganization, a similar finding to previous cognitive control studies utilizing response selection tasks such as the AX-CPT (Blanchard and Neale, 1994; Barch et al., 2003; Yoon et al., 2008). These results demonstrate that SZ does not involve generalized, non-specific impairments in episodic memory but, instead, specific deficits when cognitive control demands are high – analogous to findings in other cognitive domains (Lesh et al., 2011).

fMRI results paralleled behavioral findings. During the variable rule task, whether or not individuals with SZ showed PFC impairments depended upon the specific cognitive control demand and brain region being tested. When lower-level semantic processing and maintenance demands were emphasized (i.e., target versus non-target contrast), both groups successfully activated the left VLPFC, and VLPFC activation was positively correlated with retrieval success in SZ and HC. Conversely, when higher-level DLPFC control was required to overcome pre-potent encoding responses (i.e., non-target versus target contrast) and guide encoding toward task-appropriate responses (i.e., encode targets), resulting left DLPFC activation was reduced in SZ, and DLPFC activation correlated with better retrieval success only in HC. This lack of correlation between DLPFC activation and performance in SZ may be counter-intuitive as one might expect the DLPFC to contribute to performance even if the magnitude of activation was attenuated. However, a recent electrophysiology study of working memory (Leonard et al., 2013) found a similar mismatch in performance correlations between HC and SZ, suggesting that the two groups were employing different neural mechanisms to accomplish task performance.

To our knowledge, this is the first fMRI study to dissociate performance-related patient deficits in DLPFC versus VLPFC function during episodic memory encoding. However, relatively preserved VLPFC and disrupted DLPFC function in people with SZ was observed in previous working memory and response selection tasks at both the individual study level (e.g., Barch et al., 2001; Perlstein et al., 2001; MacDonald et al., 2005), and at the meta-analytic level (Glahn et al., 2005; Minzenberg et al., 2009), supporting the hypothesis that anatomically specific dysfunction of the DLPFC may be a central deficit underlying dysfunction across a range of cognitive domains in schizophrenia (Lesh et al., 2011).

In summary, these results suggest that individuals with SZ have difficulties with episodic encoding of word lists when DLPFC mediated cognitive control processes are required to flexibly adjust encoding strategies to match current environmental demands. In contrast, when contextual demands are less variable, and semantic encoding instructions are provided, people with SZ are as successful as HC at engaging VLPFC control processes to promote episodic memory for item information. This VLPFC activity may play a compensatory role in episodic memory in schizophrenia, as has been suggested in several functional connectivity studies finding increased VLPFC and decreased DLPFC connectivity with the medial temporal lobe in patient samples (Meyer-Lindenberg et al., 2005; Wolf et al., 2007). However, given the variable nature of our learning environments, new treatments designed to facilitate DLPFC control processes will be needed to fully restore episodic memory in schizophrenia. It is also important to note that the current item-specific memory paradigm does not address the more severe relational memory deficits experienced by individuals with schizophrenia (Heckers, 2001; Preston et al., 2005; Lepage et al., 2006; Hannula et al., 2010; Williams et al., 2010), These relational memory deficits remain relatively unresponsive to current treatments and deserving of further study.

## Author Contributions

JR took primary responsibility for running the study, data analysis and manuscript preparation, and worked together with CR and CC on study design. CR and CC shared responsibility for study design, data analysis and manuscript preparation. JP assisted with data collection and data analysis. TL and TN assisted with clinical intake and assessment procedures. MB, AK, DL, SL, MS, and RY all assisted with discussion of task design and analysis of study data. All authors provided input on final manuscript preparation.

## Conflict of Interest Statement

The authors declare that the research was conducted in the absence of any commercial or financial relationships that could be construed as a potential conflict of interest.

## Footnotes

1 http://websites.psychology.uwa.edu.au/school/MRCDatabase/uwa_mrc.htm

2 http://www.voxbo.org
