# Supplementary figures and images for: Cognitive Control of Episodic Memory in Schizophrenia: Differential Role of Dorsolateral and Ventrolateral Prefrontal Cortex
Source: Front Hum Neurosci. 2015 Nov 10;9:604. doi: 10.3389/fnhum.2015.00604 (PMC4639631; doi:10.3389/fnhum.2015.00604)

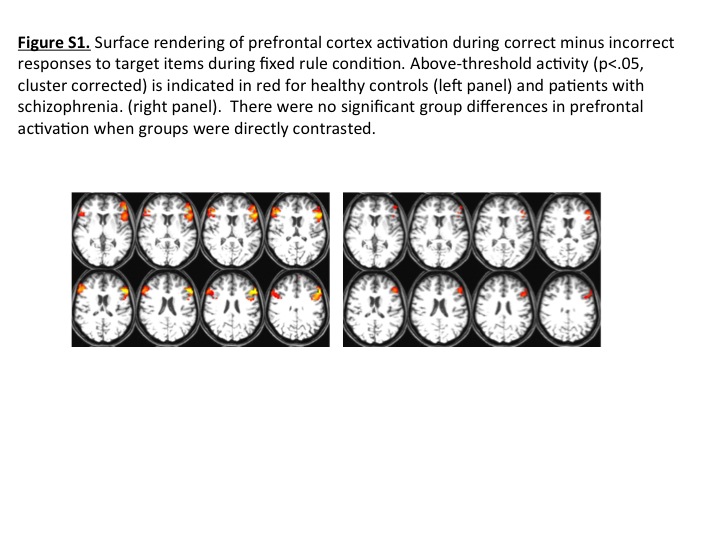

Supplement: Supplementary file 1 [file Image_1.JPEG]
